# 5-(4,5-Diiodo-1,3-dithiol-2-yl­idene)-4′,5′-bis­(methyl­sulfan­yl)-2,2′-bi-1,3-dithiole-4(5*H*)-thione

**DOI:** 10.1107/S1600536809042573

**Published:** 2009-10-23

**Authors:** Kazumasa Ueda, Kenji Yoza

**Affiliations:** a Division of Applied Science and Fundamental Engineering, Faculty of Engineering, Shizuoka University, Johoku 3-5-1, Hamamatsu, Shizuoka 432-8561, Japan; b Bruker AXS Co. Ltd., Moriya-cho 3-9, Kanagawa-ku, Kanagawa, Kanagawa 221-0022, Japan

## Abstract

The mol­ecular skeleton of the title mol­ecule, C_11_H_6_I_2_S_9_, is nearly planar [maximum deviation 0.052 (3) Å] except for the two methyl groups. In the crystal, mol­ecules related by translation along *b* axis are associated into columns through π–π inter­actions between the five-membered rings, with a centroid–centroid distance of 3.593 (5) Å. Inter­action between adjacent columns is accomplished by short S⋯I contacts of 3.2099 (4) Å.

## Related literature

For background to tetra­thia­fulvalenothio­quinone-1,3-dithiol­emethide derivatives, see: Iwamatsu *et al.* (2000 ▶); Wang *et al.* (2005 ▶, 2007 ▶); Hiraoka *et al.* (2005 ▶); Fujiwara *et al.* (2006 ▶, 2007 ▶). For details of the synthesis, see Iwamatsu *et al.* (1999 ▶). For inter­molecular S⋯I contacts, see: Ahlsen & Strømme (1974 ▶); Herbstein & Schwortzer (1984 ▶); Freemanm *et al.* (1988 ▶); Bigoli *et al.* (1996 ▶). For van der Waals radii, see: Bondi (1964 ▶).
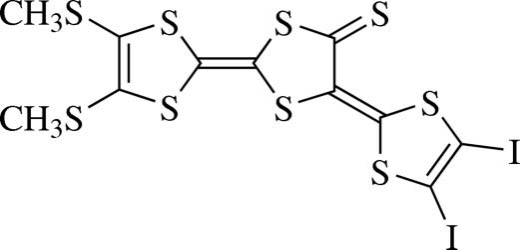


## Experimental

### 

#### Crystal data

C_11_H_6_I_2_S_9_
*M* *_r_* = 680.50
Monoclinic, 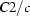

*a* = 29.540 (7) Å
*b* = 5.3543 (13) Å
*c* = 25.163 (6) Å
β = 103.544 (3)°
*V* = 3869.2 (16) Å^3^
*Z* = 8
Mo *K*α radiation
μ = 4.21 mm^−1^
*T* = 93 K
0.55 × 0.18 × 0.01 mm

#### Data collection

Bruker APEXII CCD area-detector diffractometer
Absorption correction: multi-scan (*SADABS*; Sheldrick, 1996 ▶) *T*_min_ = 0.205, *T*_max_ = 0.979
10568 measured reflections
4387 independent reflections
3536 reflections with *I* > 2σ(*I*)
*R*_int_ = 0.040

#### Refinement

*R*[*F* ^2^ > 2σ(*F* ^2^)] = 0.038
*wR*(*F* ^2^) = 0.090
*S* = 1.07
4387 reflections
201 parameters
H-atom parameters constrained
Δρ_max_ = 3.45 e Å^−3^
Δρ_min_ = −2.31 e Å^−3^

### 

Data collection: *APEX2* (Bruker, 2006 ▶); cell refinement: *SAINT* (Bruker, 2004 ▶); data reduction: *SAINT*; program(s) used to solve structure: *SHELXS97* (Sheldrick, 2008 ▶); program(s) used to refine structure: *SHELXL97* (Sheldrick, 2008 ▶); molecular graphics: *Mercury* (Macrae *et al*., 2006 ▶); software used to prepare material for publication: *XCIF* (Bruker, 2001 ▶).

## Supplementary Material

Crystal structure: contains datablocks I, global. DOI: 10.1107/S1600536809042573/cv2627sup1.cif

Structure factors: contains datablocks I. DOI: 10.1107/S1600536809042573/cv2627Isup2.hkl

Additional supplementary materials:  crystallographic information; 3D view; checkCIF report

## Figures and Tables

**Fig. 1.**
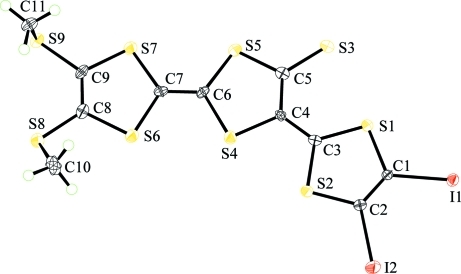
The molecular structure of (I) showing atom labelling and 50% probability displacement ellipsoids for non H-atoms.

**Fig. 2.**
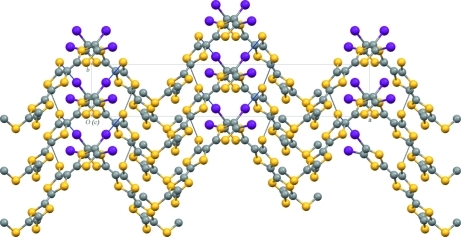
Projection of the crystal packing in (I) down the ab plane. The S···S (black) and S···I (blue) contacts are shown with dashed lines. H atoms are omitted for clarity.

**Fig. 3.**
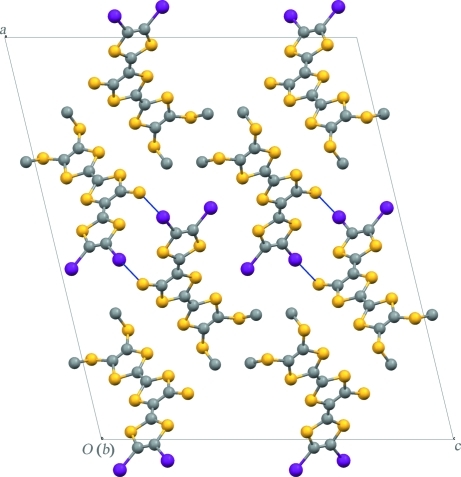
Projection of the crystal packing in (I) down the ac plane. The S···I (blue) contacts are shown with dashed lines. H atoms are omitted for clarity.

## supplementary crystallographic information

### Comment

Charge transfer (CT) complexes of new donor molecules featuring a skeleton of tetrathiafulvalenothioquinone-1,3-dithiolemethide with magnetic metal ions are used for the preparation of magnetic molecular conductors, especially ferromagnetic semiconductors and metals (Wang *et al.*, 2005, 2007; Hiraoka *et al.*, 2005; Fujiwara *et al.*, 2006, 2007). In the CT salts of an ethylendithiotetrathiafulvalenothioquinone-1,3-dithiolemethide donor with CuBr_2_, the Cu atom (a Lewis acid) of CuBr_2_ is bound to the S atom (a Lewis base) of a C=S group in the donor to form a new type of π/d molecular system (Iwamatsu *et al.*, 2000). The introduction of Lewis acids, such as iodine atoms, as substituents in the molecular skeleton is expected to enhance intermolecular interaction through the formation of S···I contacts. These contacts are of special interest in these structures as they may increase the dimensionality of aggregation in the solid-state. In this context, the crystal structure of the title compound, (I), was investigated.

The molecular framework of (I), Fig. 1, except for two methylthio groups, is almost planar [maximum deviation 0.052 (3) Å]. The displacements of atoms S8, S9, I1 and I2 relative to the plane of the skeleton are -0.164 (3), -0.151 (3), 0.164 (3) and 0.277 (3) Å, respectively. The torsion angles of the two methylthio groups are -136.95° for C10—S8—C8—C9 and 80.97° for C11—S9—C9—C8. In the crystal structure, two different arrangements of the molecules are present. One arrangement has a dihedral angle of 48.41° to the *ac* plane, while the other has a dihedral angle of 131.51° to the *ac *plane. As a result, the molecules are stacked in the same orientations to form one-dimensional columns along the [110] and [1–10] directions (Fig. 2). Although the weak interaction between stacked molecules in the columns is accomplished through contacts between different sulfur atoms [S3···S5^i^ = 3.5916 (6) Å; symmetry code: (i) +*x*, 1 + *y*, +*z*] is shorter than the sum of van der Waals radii of two sulfur atoms (3.60 Å), stacked molecules are separated by interplanar distances greater than 3.54 Å and have fairly poor overlap (Bondi, 1964). However, some effective side-by-side contacts are observed between molecules of adjacent columns. The interaction between columns is accomplished by contacts between sulfur and iodine atoms [S3···I1^ii^ = 3.2099 (4) Å; symmetry code: (ii) 2 - *x*, -1 + *y*, 1/2 - *z*] along the *a* axis (Fig. 3). This distance is shorter than the sum of van der Waals radii of sulfur and iodine atoms (3.78 Å). In the two molecules bound by sulfur-iodine interaction, the C5—S3—I1^ii^—C1^ii^ moieties are not planar and almost linear S3—I1^ii^—C1^ii ^fragments lie roughly perpendicular to the molecular skeleton [torsion angle of -81.47° for I1^i^—S3—C5—S5 and torsion angle of 97.60° for I1^i^—S3—C5—C4], and the dihedral angle of the molecules is 83.11°. Such sulfur-iodine interactions have been observed previously (Ahlsen *et al.*,1974; Herbstein *et al.*, 1984; Freemanm *et al.*, 1988; Bigoli *et al.*, 1996).

### Experimental

Compound (I) was synthesized by a modification of the method used for the preparation of bis(methylthio)tetrathiafulvalenothioquinone-1,3-dithiolemethide (Iwamatsu *et al.*,1999). Bis(tetraethylammonium)bis(2,3-bis(methylthio)tetrathiafulvalenyl-6,7-dithiolato)zinc (269 mg, 0.258 mmol) was reacted with 4,5-diiodo-2-methylthio-1,3-dithiole-2,3-dithiolium tetrafluoroborate (535 mg, 1.10 mmol) in THF-DMF (5:1 = *v*/*v*) at room temperature under nitrogen, and stirring was carried out for 12 h. After separation of the reaction mixture by column chromatography on silica gel (eluent: CS_2_) followed by recrystallization from CS_2_/*n*-hexane, (I) was obtained as black needles in 72% yield.

### Refinement

The H atoms were geometrically positioned with C—H: 0.98 Å, and refined as riding, with *U*_iso_(H)= 1.5*U*_eq_(C). The highest residual peak [3.45 e Å^-3^] and deepest hole [-2.31 e Å^-3^] are situated 0.98 Å and 0.69 Å at atom I2, respectively.

### Crystal data

**Table:**

C_11_H_6_I_2_S_9_	*F*(000) = 2576
*M**_r_* = 680.50	*D*_x_ = 2.336 Mg m^−^^3^
Monoclinic, *C*2/*c*	Mo *K*α radiation, λ = 0.71073 Å
Hall symbol: -C 2yc	Cell parameters from 2871 reflections
*a* = 29.540 (7) Å	θ = 2.4–27.5°
*b* = 5.3543 (13) Å	µ = 4.21 mm^−^^1^
*c* = 25.163 (6) Å	*T* = 93 K
β = 103.544 (3)°	Needle, black
*V* = 3869.2 (16) Å^3^	0.55 × 0.18 × 0.01 mm
*Z* = 8	

### Data collection

**Table:**

Bruker APEXII CCD area-detector diffractometer	4387 independent reflections
Radiation source: Bruker TXS fine-focus rotating anode	3536 reflections with *I* > 2σ(*I*)
Bruker Helios multilayer confocal mirror	*R*_int_ = 0.040
Detector resolution: 8.333 pixels mm^-1^	θ_max_ = 27.5°, θ_min_ = 1.4°
φ and ω scans	*h* = −38→36
Absorption correction: multi-scan (*SADABS*; Sheldrick, 1996)	*k* = −6→6
*T*_min_ = 0.205, *T*_max_ = 0.979	*l* = −24→32
10568 measured reflections	

### Refinement

**Table:**

Refinement on *F*^2^	Primary atom site location: structure-invariant direct methods
Least-squares matrix: full	Secondary atom site location: difference Fourier map
*R*[*F*^2^ > 2σ(*F*^2^)] = 0.038	Hydrogen site location: inferred from neighbouring sites
*wR*(*F*^2^) = 0.090	H-atom parameters constrained
*S* = 1.07	*w* = 1/[σ^2^(*F*_o_^2^) + (0.028*P*)^2^ + 20.8292*P*] where *P* = (*F*_o_^2^ + 2*F*_c_^2^)/3
4387 reflections	(Δ/σ)_max_ = 0.002
201 parameters	Δρ_max_ = 3.45 e Å^−^^3^
0 restraints	Δρ_min_ = −2.31 e Å^−^^3^

### Special details

**Table:**

Geometry. All e.s.d.'s (except the e.s.d. in the dihedral angle between two l.s. planes) are estimated using the full covariance matrix. The cell e.s.d.'s are taken into account individually in the estimation of e.s.d.'s in distances, angles and torsion angles; correlations between e.s.d.'s in cell parameters are only used when they are defined by crystal symmetry. An approximate (isotropic) treatment of cell e.s.d.'s is used for estimating e.s.d.'s involving l.s. planes.
Refinement. Refinement of *F*^2^ against ALL reflections. The weighted *R*-factor *wR* and goodness of fit *S* are based on *F*^2^, conventional *R*-factors *R* are based on *F*, with *F* set to zero for negative *F*^2^. The threshold expression of *F*^2^ > σ(*F*^2^) is used only for calculating *R*-factors(gt) *etc*. and is not relevant to the choice of reflections for refinement. *R*-factors based on *F*^2^ are statistically about twice as large as those based on *F*, and *R*- factors based on ALL data will be even larger.

### Fractional atomic coordinates and isotropic or equivalent isotropic displacement parameters (Å^2^)

**Table:**

	*x*	*y*	*z*	*U*_iso_*/*U*_eq_	
C1	1.01287 (15)	1.3727 (9)	0.35298 (19)	0.0158 (10)	
C2	1.02057 (16)	1.2692 (10)	0.4024 (2)	0.0198 (11)	
C3	0.94845 (15)	1.0393 (9)	0.34447 (18)	0.0150 (10)	
C4	0.91248 (16)	0.8707 (9)	0.32900 (19)	0.0157 (10)	
C5	0.88305 (16)	0.8507 (9)	0.27608 (19)	0.0152 (10)	
C6	0.85596 (16)	0.5009 (9)	0.3338 (2)	0.0177 (10)	
C7	0.83308 (16)	0.3149 (10)	0.35177 (19)	0.0180 (11)	
C8	0.80459 (17)	−0.0037 (10)	0.4151 (2)	0.0213 (11)	
C9	0.77585 (16)	−0.0346 (10)	0.3660 (2)	0.0197 (11)	
C10	0.8099 (2)	0.0697 (12)	0.5245 (2)	0.0345 (14)	
H10A	0.8431	0.1096	0.5359	0.052*	
H10B	0.7988	0.0128	0.5562	0.052*	
H10C	0.7925	0.2190	0.5090	0.052*	
C11	0.68641 (17)	−0.0497 (11)	0.3739 (2)	0.0244 (12)	
H11A	0.6975	−0.0211	0.4132	0.037*	
H11B	0.6563	−0.1358	0.3667	0.037*	
H11C	0.6828	0.1110	0.3547	0.037*	
I1	1.052758 (10)	1.64937 (6)	0.326836 (12)	0.01695 (10)	
I2	1.075939 (14)	1.33621 (9)	0.468614 (15)	0.04003 (14)	
S1	0.96539 (4)	1.2618 (2)	0.30265 (5)	0.0163 (2)	
S2	0.98132 (4)	1.0369 (3)	0.41150 (5)	0.0202 (3)	
S3	0.88601 (4)	1.0394 (2)	0.22343 (5)	0.0186 (3)	
S4	0.90284 (4)	0.6524 (2)	0.37793 (5)	0.0196 (3)	
S5	0.84272 (4)	0.6130 (2)	0.26660 (5)	0.0194 (3)	
S6	0.84983 (4)	0.2173 (3)	0.42068 (5)	0.0217 (3)	
S7	0.78627 (4)	0.1484 (3)	0.31171 (5)	0.0200 (3)	
S8	0.80104 (5)	−0.1743 (3)	0.47356 (5)	0.0231 (3)	
S9	0.72837 (4)	−0.2411 (3)	0.34965 (5)	0.0200 (3)	

### Atomic displacement parameters (Å^2^)

**Table:**

	*U*^11^	*U*^22^	*U*^33^	*U*^12^	*U*^13^	*U*^23^
C1	0.013 (2)	0.020 (3)	0.015 (2)	−0.0029 (19)	0.0032 (18)	−0.002 (2)
C2	0.018 (2)	0.023 (3)	0.017 (2)	−0.004 (2)	−0.0002 (19)	0.002 (2)
C3	0.015 (2)	0.017 (3)	0.012 (2)	0.0032 (19)	0.0015 (18)	−0.0001 (19)
C4	0.015 (2)	0.015 (3)	0.017 (2)	0.0032 (19)	0.0038 (18)	0.004 (2)
C5	0.017 (2)	0.016 (3)	0.015 (2)	0.0061 (19)	0.0073 (18)	−0.0025 (19)
C6	0.017 (2)	0.015 (3)	0.020 (3)	−0.0027 (19)	0.0026 (19)	−0.004 (2)
C7	0.017 (2)	0.020 (3)	0.016 (2)	0.000 (2)	0.0033 (19)	−0.003 (2)
C8	0.018 (2)	0.020 (3)	0.026 (3)	−0.004 (2)	0.007 (2)	−0.001 (2)
C9	0.013 (2)	0.020 (3)	0.027 (3)	−0.002 (2)	0.0050 (19)	−0.004 (2)
C10	0.047 (4)	0.035 (4)	0.024 (3)	−0.007 (3)	0.015 (3)	−0.002 (3)
C11	0.018 (2)	0.029 (3)	0.026 (3)	−0.001 (2)	0.005 (2)	−0.004 (2)
I1	0.01587 (16)	0.01857 (18)	0.01695 (17)	−0.00209 (12)	0.00491 (12)	−0.00065 (13)
I2	0.0346 (2)	0.0579 (3)	0.0204 (2)	−0.0239 (2)	−0.00805 (16)	0.01043 (18)
S1	0.0161 (6)	0.0184 (6)	0.0143 (6)	−0.0014 (5)	0.0033 (4)	0.0016 (5)
S2	0.0195 (6)	0.0269 (7)	0.0133 (6)	−0.0053 (5)	0.0019 (5)	0.0029 (5)
S3	0.0198 (6)	0.0193 (7)	0.0160 (6)	0.0023 (5)	0.0027 (5)	0.0014 (5)
S4	0.0223 (6)	0.0212 (7)	0.0153 (6)	−0.0046 (5)	0.0046 (5)	0.0002 (5)
S5	0.0188 (6)	0.0194 (7)	0.0185 (6)	−0.0034 (5)	0.0014 (5)	−0.0020 (5)
S6	0.0208 (6)	0.0254 (8)	0.0182 (6)	−0.0065 (5)	0.0031 (5)	−0.0005 (5)
S7	0.0200 (6)	0.0215 (7)	0.0179 (6)	−0.0040 (5)	0.0033 (5)	−0.0012 (5)
S8	0.0254 (7)	0.0233 (7)	0.0202 (7)	−0.0050 (5)	0.0044 (5)	0.0014 (5)
S9	0.0182 (6)	0.0207 (7)	0.0214 (6)	−0.0037 (5)	0.0052 (5)	−0.0039 (5)

### Geometric parameters (Å, °)

**Table:**

C1—C2	1.331 (7)	C7—S7	1.752 (5)
C1—S1	1.758 (5)	C7—S6	1.767 (5)
C1—I1	2.093 (5)	C8—C9	1.336 (7)
C2—S2	1.751 (5)	C8—S8	1.755 (5)
C2—I2	2.074 (5)	C8—S6	1.766 (5)
C3—C4	1.379 (7)	C9—S9	1.758 (5)
C3—S2	1.738 (4)	C9—S7	1.766 (5)
C3—S1	1.739 (5)	C10—S8	1.806 (6)
C4—C5	1.414 (6)	C10—H10A	0.9800
C4—S4	1.769 (5)	C10—H10B	0.9800
C5—S3	1.685 (5)	C10—H10C	0.9800
C5—S5	1.721 (5)	C11—S9	1.820 (5)
C6—C7	1.340 (7)	C11—H11A	0.9800
C6—S5	1.750 (5)	C11—H11B	0.9800
C6—S4	1.758 (5)	C11—H11C	0.9800
			
C2—C1—S1	117.7 (4)	C8—C9—S9	126.4 (4)
C2—C1—I1	127.1 (4)	C8—C9—S7	117.3 (4)
S1—C1—I1	115.2 (2)	S9—C9—S7	116.2 (3)
C1—C2—S2	116.4 (4)	S8—C10—H10A	109.5
C1—C2—I2	127.5 (4)	S8—C10—H10B	109.5
S2—C2—I2	116.1 (3)	H10A—C10—H10B	109.5
C4—C3—S2	119.2 (4)	S8—C10—H10C	109.5
C4—C3—S1	126.1 (4)	H10A—C10—H10C	109.5
S2—C3—S1	114.7 (3)	H10B—C10—H10C	109.5
C3—C4—C5	125.4 (5)	S9—C11—H11A	109.5
C3—C4—S4	118.4 (4)	S9—C11—H11B	109.5
C5—C4—S4	116.2 (4)	H11A—C11—H11B	109.5
C4—C5—S3	124.2 (4)	S9—C11—H11C	109.5
C4—C5—S5	116.1 (4)	H11A—C11—H11C	109.5
S3—C5—S5	119.8 (3)	H11B—C11—H11C	109.5
C7—C6—S5	124.4 (4)	C3—S1—C1	95.1 (2)
C7—C6—S4	121.0 (4)	C3—S2—C2	96.0 (2)
S5—C6—S4	114.6 (3)	C6—S4—C4	95.5 (2)
C6—C7—S7	125.2 (4)	C5—S5—C6	97.4 (2)
C6—C7—S6	120.2 (4)	C8—S6—C7	95.0 (2)
S7—C7—S6	114.6 (3)	C7—S7—C9	95.4 (2)
C9—C8—S8	124.1 (4)	C8—S8—C10	101.0 (3)
C9—C8—S6	117.5 (4)	C9—S9—C11	97.8 (2)
S8—C8—S6	118.4 (3)		
